# Cerebellar Ataxia Caused by Central Nervous System Tuberculosis With Pulmonary Tuberculosis: A Case Report

**DOI:** 10.7759/cureus.33493

**Published:** 2023-01-07

**Authors:** Yusuke Saishoji, Hideki Mori, Yasumori Izumi

**Affiliations:** 1 General Internal Medicine, National Hospital Organization Nagasaki Medical Center, Ōmura, JPN

**Keywords:** case report, ataxia, infection, central nervous system, tuberculosis

## Abstract

Central nervous system (CNS) tuberculosis (TB) remains a serious disease with high morbidity and mortality but is often difficult to diagnose owing to less sensitive microbiological techniques. Herein, we present a case where the main complaint was staggering gait; however, the patient was diagnosed with CNS TB associated with pulmonary TB. A woman in her 70s was admitted to our hospital with a two-month history of progressive ataxia. Cerebrospinal fluid examination showed an elevated lymphocyte count; however, cranial imaging studies did not show significant findings. However, we performed positron emission tomography-computed tomography imaging owing to suspicions of paraneoplastic syndrome, which showed substantial ^18^F-fluorodeoxyglucose accumulation in the lungs. A subsequent bronchoscopy exam led to a pulmonary TB diagnosis for which the patient was treated, and the patient’s symptoms fully resolved. Finally, we diagnosed ataxia due to CNS TB with pulmonary TB after excluding other causes of ataxia and because of a lymphocyte-predominant increase of cells in the spinal fluid. Thus, TB infection should be considered in cases of cerebellar ataxia of unknown etiology.​​

## Introduction

Tuberculosis (TB) mainly presents as a pulmonary disease but can also affect other organs, causing extrapulmonary TB. Central nervous system (CNS) TB is the most destructive form, with high disability and mortality rates; thus, early diagnosis and treatment are critical [1]. However, CNS TB diagnoses are difficult because the clinical features are non-specific, and the laboratory tests are insensitive. For example, it cannot be ruled out by negative spinal fluid cultures, smears, or nuclear amplification tests [2,3]. Therefore, TB infection should also be considered when encountering neurological symptoms of an unclear etiology. Herein, we report our experience with a case of cerebellar ataxia caused by CNS TB with pulmonary TB.

## Case presentation

A woman in her 70s was admitted to our hospital with a two-month history of progressive ataxia. The patient had a history of constipation for 10 years and osteoporosis for 15 years that was treated with magnesium oxide 990mg/day and raloxifene 60mg/day. The patient had no history or contact with TB. The first time the patient experienced symptoms was during a walk when they felt a leaning sensation even though they intended to walk in a straight line. Furthermore, the patient experienced several falls in well-lit areas, with no reduction or exacerbation in the dark. Finally, the patient had no history of convulsive seizures or cognitive decline and no history of alcoholism or substance abuse, unemployment, immigration, or imprisonment.

On admission, the patient’s Glasgow Coma Scale was E4V5M6. Furthermore, their blood pressure, heart rate, peripheral arterial oxygen saturation (ambient air), respiratory rate, and body temperature were 102/56 mmHg, 66 beats/min, 98%, 12 breaths/min, and 37.2°C, respectively. The patient's body mass index was 19.2. The patient's respiratory system examination was normal with no crackles, normal S1 and S2, no murmur or gallops were heard, the abdomen was soft, and no tenderness or organomegaly was noted. A neurological examination of the cranial nerves did not reveal abnormalities, and the motor examination was normal. The sensory examination revealed intact pinprick sensation, temperature test, proprioception, and vibration in all extremities. Babinski reflex and Romberg test results were negative. However, the patient had a wide-based gait with a tandem gait abnormality and bilateral dysmetria based on finger-nose-finger testing. Diadochokinesis slowness was also observed bilaterally.

The blood tests revealed the following findings: 4,600/μL white blood cell count with 2,760/μL neutrophils, 12.6g/dL hemoglobin, 238,000/μL platelets, 6.0mm/hr erythrocyte sedimentation rate, normal vitamin B1, B12, and E and folic acid levels and no syphilis antibodies. The patient’s thyroid function was normal; however, the serum tested positive for anti-thyroid peroxidase antibodies at 125IU/mL. We considered Hashimoto’s encephalopathy as a differential diagnosis, but the serum anti-NH2 terminal of alpha-enolase (i.e., NAE) antibody test was negative. Furthermore, anti-amphiphysin (i.e., AMPH) antibody, anti-CV2 antibody, anti-paraneoplastic antigen MA2 (i.e., PNMA2) antibody, anti-Ri antibody, anti-Yo antibody, anti-Hu antibody, anti-recoverin antibody, anti-SRY-related HMG-box gene 1 (i.e., SOX1) antibody, anti-titin antibody, anti-zic4 antibody, anti-glutamic acid decarboxylase 65 (i.e., GAD65) antibody, and anti-Tr/delta/notch-like epidermal growth factor-related receptor (i.e., DNER) antibody tests performed with serum using the EUROLINE Paraneoplastic Neurological Syndromes 12 Ag (Euroimmun, Lübeck, Germany) for tumor-associated syndromes were negative. A screening of rat brain slices was not performed.

**Figure 1:**
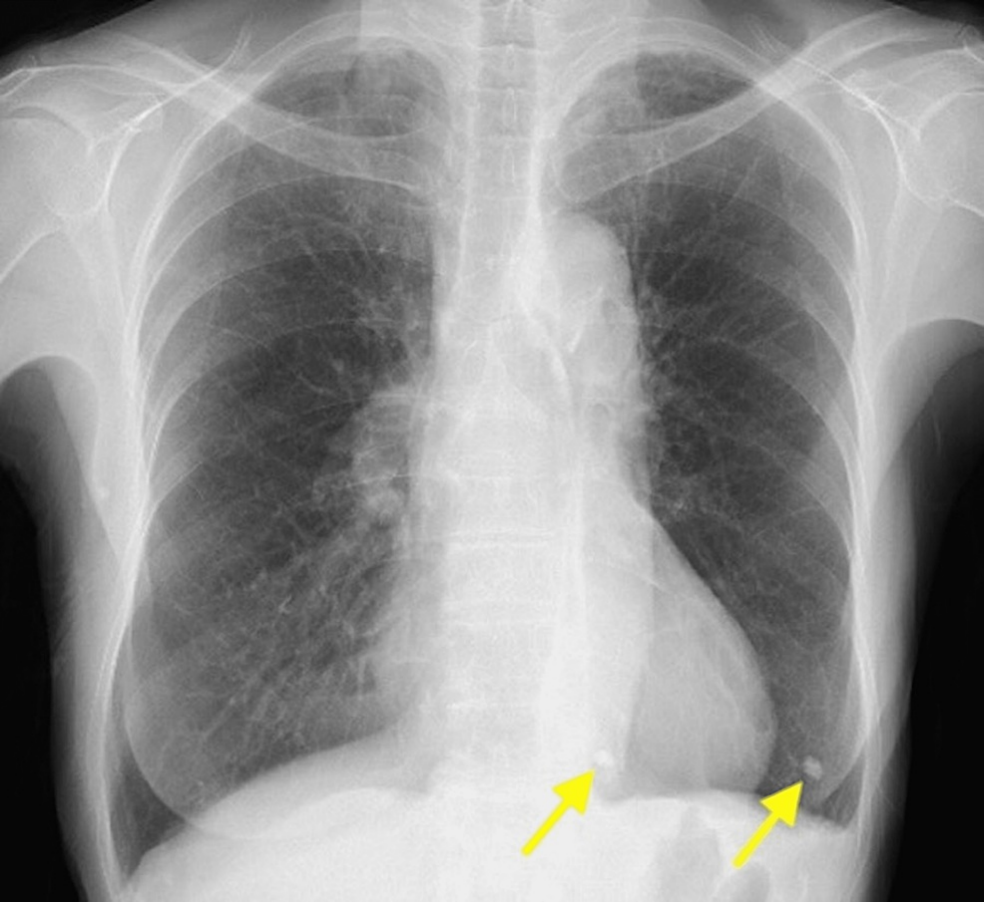
Chest X-ray image Scattered calcified lesions in the left lower lung.

**Figure 2:**
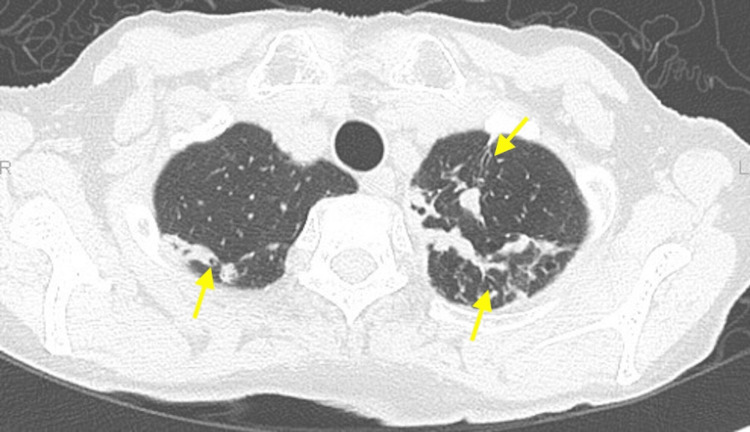
Chest computed tomography scan Chest computed tomography scan on admission showing dilated bronchioles surrounded by granular, chordate, and plaque shadows (yellow arrows) in the bilateral lung apexes.

**Figure 3:**
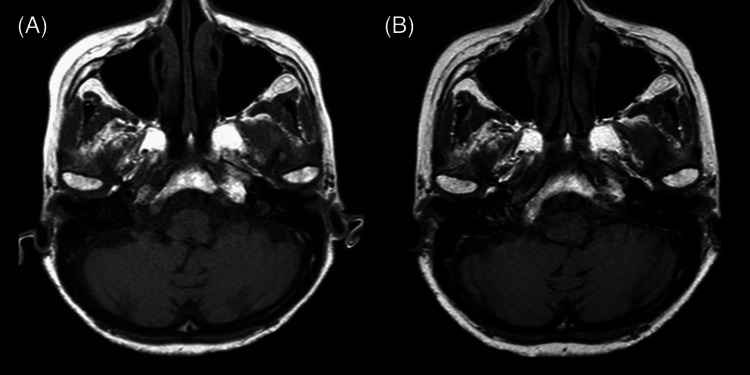
Magnetic resonance images T1-weighted magnetic resonance images at the upper levels of the cerebellum on admission (A) and two years after tuberculosis treatment (B). Neither image shows swelling, atrophy of the cerebellum, or other abnormal signals.

**Figure 4:**
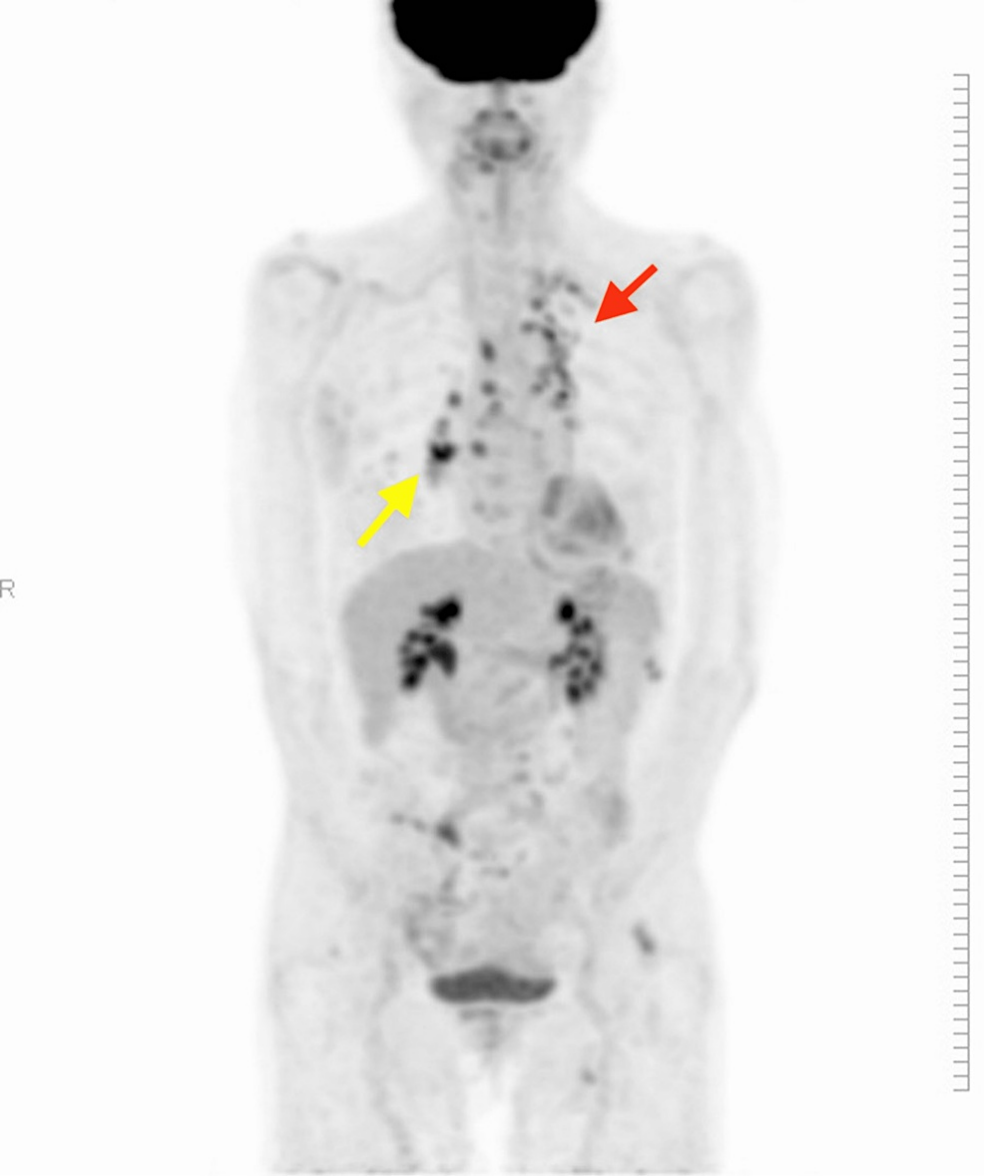
Positron emission tomography-computed tomography imaging Positron emission tomography-computed tomography imaging shows ^18^F-fluorodeoxyglucose accumulation in the hilar lymph nodes (yellow arrow) and the left upper lung (red arrow).

Upper and lower gastrointestinal endoscopy, mammography, pelvic magnetic resonance imaging (MRI), and whole-body contrast-enhanced computed tomography (CT) did not identify malignant tumors. The chest X-ray (Figure 1) showed scattered calcified lesions in the left lower lung, but no other significant findings were evident. However, whole-body contrast-enhanced CT (Figure 2) showed dilated bronchioles with granular, chordate, and plaque shadows in the bilateral lung apexes. Cerebrospinal fluid (CSF) examinations showed an elevated cell count of 13 cells/μL (neutrophils: 21%, lymphocytes: 68%, and histiocytes: 11%) but normal protein, glucose, and adenosine deaminase levels, and the oligoclonal band were negative. Polymerase chain reaction tests for antimicrobials, varicella-zoster virus, and herpes simplex virus (HSV) were negative. The cytological diagnosis was Class I with Papanicolaou staining, and no atypical cells were identified. Bacterial culture, acid-fast bacilli culture, and *Mycobacterium tuberculosis* polymerase chain reaction tests with CSF were negative, as were urine and sputum mycobacterium culture tests. Head CT and brain MRI scans were normal (Figure 3), but positron emission tomography-CT imaging (Figure 4) showed significant ^18^F-fluorodeoxyglucose accumulation in the hilar lymph nodes and left lung. A subsequent bronchoscopy exam led to a pulmonary TB diagnosis with *Mycobacterium tuberculosis* by smear and culture testing of bronchoalveolar lavage fluid.

Our differential diagnosis included acquired and sporadic degenerative ataxias, such as alcoholic cerebellar degeneration, other toxic causes, paraneoplastic cerebellar degeneration, meningitis, encephalopathy, and multiple system atrophy. However, these were all ruled out, except for meningitis, based on the lack of excessive alcohol consumption or drug use in the patient’s history, the absence of brain atrophy and abnormal signal regions on brain MRI, and the negative malignancy screening results. In addition, the elevated cell count in spinal fluid potentially indicated CNS TB as the cause of cerebellar ataxia. Although no bacteria were detected in the spinal fluid culture, the patient had active pulmonary TB, which could affect the CNS. Therefore, we decided to administer pulmonary TB treatment.

The patient underwent two months of treatment with isoniazid (5mg/kg/day), rifampin (10mg/kg/day), ethambutol (15mg/kg/day), and pyrazinamide (25mg/kg/day), followed by four months of isoniazid and rifampin. Drug resistance testing for *Mycobacterium tuberculosis* was performed; however, no resistance was identified against all drugs. Corticosteroids were not prescribed. A physical therapist provided physical therapy during the hospital stay. Within a few weeks of starting treatment, the patient’s symptoms resolved, as demonstrated in Video 1, which compares their walking before and after pulmonary TB treatment. In this video, the patient presents with a slight camptocormia and decreased spontaneous movements of the right upper limb. However, finding characteristics of Parkinson's disease, such as tremors, immobility, rigidity, and postural dysreflexia, were not observed. Five years have passed since administering the treatment without symptom recurrence.

**Video 1:** The patient’s gait before and after pulmonary tuberculosis treatment. After treatment, the ataxic gait disappeared, and the patient could walk without a handrail.

Finally, we diagnosed cerebellar ataxia due to TB infection as a diagnosis of exclusion because all neurological abnormalities, including gait, improved with only TB treatment. The pulmonary TB diagnosis and the elevated lymphocyte-predominant cell count in the spinal fluid examination also suggested CNS TB was caused by *Mycobacterium tuberculosis*.

## Discussion

CNS TB is a devastating form of TB that causes significant morbidity and mortality, even with appropriate therapy [4]. Despite molecular diagnostic technique advances, CNS TB diagnoses remain primarily dependent on less sensitive microbiological techniques, making CNS TB challenging to diagnose [5]. Cases of cerebellar ataxia due to CNS TB have been reported [6] following cases of cerebellar ataxia due to bacterial meningitis [7]. Specifically, there have been reports of cerebellar ataxia symptoms in patients with pulmonary TB (Table 1). The present case is unique in that it only showed elevated cell counts in the CSF examinations. *Mycobacterium tuberculosis* was not identified on CSF culture, and the brain MRI scans were normal. Conversely, previous reports have identified *Mycobacterium tuberculosis* in CSF samples and abnormal findings on brain MRI scans. Thus, the cerebellar ataxia diagnosis, in this case, was very difficult owing to the lack of findings.

**Table 1:** Cases of cerebellar ataxia with pulmonary TB TB: tuberculosis

Author	Sex	Age	Symptoms	Infected organ
Khadir et al. (2013) [6]	Female	38	Difficulty in walking, ataxia, and weight loss	Pulmonary, cerebral
Ashraf et al. (2011) [8]	Male	8	Unsteadiness of gait, tremor of hands, and spontaneous, intermittent, nonrhythmic jerking of lower limbs and trunk	Pulmonary, cerebral
Velásquez-Rimachi et al. (2020) [9]	Female	21	Persistent headaches concomitant with nausea, vomiting, dizziness, and ataxia	Pulmonary, cerebral
Batool et al. (2022) [10]	Female	25	Headache and intractable vomiting	Pulmonary, cerebral, adrenal
Er et al. (2018) [11]	Male	53	Dizziness, nausea, and vomiting	Pulmonary, cerebral

Cerebellar ataxia is characterized by uncoordinated movements and instability due to cerebellum dysfunction. Furthermore, clinical examinations generally indicate gait disorders, such as imbalance, staggering and tandem gait difficulties, upper and lower limb dysmetria and dysdiadochokinesia, hypotonia, cerebellar dysarthria, and saccadic ocular pursuit [12]. Cerebellar ataxia has a variety of etiologies, including acquired, hereditary, and sporadic degenerative ataxias [13]. Additionally, infections may directly or indirectly affect cerebellar function, and the resulting inflammation can be viral or bacterial-induced or related to immune mechanisms triggered by the infection [14]. Therefore, this case required a careful exclusion diagnosis.

In considering the pathophysiology of this case, the patient’s medical history should also be noted. TB infection can cause constipation [15] and osteoporosis [16]. The patient had a history of constipation and osteoporosis for more than 10 years, and inflammatory findings in the bilateral pulmonary apex on chest CT scans suggested that the patient had prior pulmonary TB. From a unitary point of view, it is thought that the patient had a background of old pulmonary TB, and the infection became apparent due to advanced age, resulting in CNS involvement. Furthermore, cerebellar ataxia did not improve until the patient was treated for TB, and the symptoms improved only after TB treatment without steroid therapy supporting the suspicion that pulmonary TB had affected the CNS. Other causes, especially oral medications, may have contributed to the ataxia. The possibility that the two medications used for these diseases were associated with ataxic symptoms cannot be ruled out; however, their involvement in the pathogenesis is considered very limited because there was no discontinuation or change in medications before or after treatment for cerebellar ataxia, and there was no remission or worsening of symptoms. The other causes of ataxia, such as acquired and sporadic degenerative ataxias, including alcoholic cerebellar degeneration, other toxic causes, paraneoplastic cerebellar degeneration, encephalopathy, and multiple system atrophy, are possible differential diagnoses, however, the history, physical examination, laboratory results, and imaging studies were negative. Immune-mediated cerebellar ataxia was ruled out owing to the lack of specific antibodies detected and the increased lymphocyte-predominant cell count in the spinal fluid. Nonetheless, in this case, clearly distinguishing meningitis from encephalitis was challenging because the patient had a CNS infection with clinical features of meningeal and parenchymal disease.

In this case, *Mycobacterium tuberculosis* was not detected in the spinal fluid, which is the gold standard, highlighting the difficulty in diagnosing CNS TB. Various authors have also reported the difficulty of isolating *Mycobacterium tuberculosis* by microscopy, molecular diagnosis, and culture [2,17,18]. Therefore, empiric antimicrobial treatment is considered when difficulties related to *Mycobacterium tuberculosis* infection diagnoses arise. Our patient was treated for pulmonary TB for six months, although the treatment recommendation for CNS TB is a minimum of 10 months [19]. However, a six-month regimen has been evaluated as an alternative option for treating CNS TB [20].

## Conclusions

This case is valuable since it indicates *Mycobacterium tuberculosis* involvement is possible when the etiology of ataxia is difficult to identify. *Mycobacterium tuberculosis* involvement may be difficult to prove; however, it is important to consider in cases of CNS symptoms where the etiology is unidentifiable. We hope that more reports of similar cases will deepen our understanding of this unusual presentation.
